# Antiamoebic Activities of Indolocarbazole Metabolites Isolated from *Streptomyces sanyensis* Cultures

**DOI:** 10.3390/md17100588

**Published:** 2019-10-17

**Authors:** Luis Cartuche, María Reyes-Batlle, Ines Sifaoui, Iñigo Arberas-Jiménez, José E. Piñero, José J. Fernández, Jacob Lorenzo-Morales, Ana R. Díaz-Marrero

**Affiliations:** 1 Instituto Universitario de Bio-Orgánica Antonio González (IUBO AG), Centro de Investigaciones Biomédicas de Canarias (CIBICAN), Universidad de La Laguna (ULL), Avda. Astrofísico F. Sánchez 2, 38206 La Laguna, Tenerife, Spain; lecartuche@utpl.edu.ec (L.C.); jjfercas@ull.edu.es (J.J.F.); 2 Departamento de Química y Ciencias Exactas, Sección Química Básica y Aplicada, Universidad Técnica Particular de Loja (UTPL), San Cayetano alto s/n, A.P. 1101608 Loja, Ecuador; 3 Instituto Universitario de Enfermedades Tropicales y Salud Pública de Islas Canarias, Departamento de Obstetricia y Ginecología, Pediatría, Medicina Preventiva y Salud Pública, Toxicología, Medicina Legal y Forense y Parasitología, Universidad de La Laguna, Avda. Astrofísico F. Sánchez s/n, 38206 La Laguna, Tenerife, Spain; mreyesba@ull.edu.es (M.R.-B.); isifaoui@ull.edu.es (I.S.); alu0101283227@ull.edu.es (I.A.-J.); jpinero@ull.edu.es (J.E.P.); 4 Departamento de Química Orgánica, Universidad de La Laguna (ULL), Avda. Astrofísico F. Sánchez, 2, 38206 La Laguna, Tenerife, Spain

**Keywords:** indolocarbazole, staurosporine, *Streptomyces sanyensis*, antiparasitic activities, *Acanthamoeba*

## Abstract

Indolocarbazoles are a family of natural alkaloids characterized by their potent protein kinase and topoisomerase I inhibitory activity. Among them, staurosporine (**1**) has exhibited promising inhibitory activity against parasites. Based on new insights on the activity and mechanism of action of STS in *Acanthamoeba* parasites, this work reports the isolation, identification, and the anti-*Acanthamoeba* activity of the minor metabolites 7-oxostaurosporine (**2**), 4′-demethylamino-4′-oxostaurosporine (**3**), and streptocarbazole B (**4**), isolated from cultures of the mangrove strain *Streptomyces sanyensis.* A clear correlation between the antiparasitic activities and the structural elements and conformations of the indolocarbazoles **1**–**4** was observed. Also, the study reveals that 7-oxostaurosporine (**2**) affects membrane permeability and causes mitochondrial damages on trophozoites of *A. castellanii* Neff.

## 1. Introduction

Indolocarbazoles (ICZs) are a large family of alkaloid natural compounds characterized by their protein kinase, particularly tyrosine kinase, inhibitory activity. These enzymes are responsible for regulating essential aspects of cell metabolism including cell cycle progression; consequently, ICZs exert potent cytotoxic effect on cancer cells [1,2,3]. 

ICZs have been isolated from several sources including bacteria, slime molds, cyanobacteria, and marine invertebrates [1]. The first isolated compound, staurosporine (STS), was obtained from a culture of a soil actinomycete (*Streptomyces staurosporeus*) in 1977 [4] at the Kitasato Institute (Mizusawa City, Japan) and nine years later, its mechanism of action was discovered through non-selective protein kinase inhibition [5,6]. Related compounds demonstrated the same effect, thus opening the route for the discovery of a new class of antincancer drugs [7,8]. In addition, two synthetic derivatives of the ICZ antibiotic, K252a, were proven to be potent inducers of DNA cleavage complex by interacting with topoisomerase I, increasing the interest of pharmaceutical industries in this family of compounds [9].

STS has exhibited promising inhibitory activity against parasites [10,11,12]; since protein kinases (PKs) play critical roles in the growth and mechanism of infection of some parasites such as *Trypanosoma* and *Leishmania* spp., and parasitic PKs differ from their mammal counterparts, these antibiotics result attractive for the development of specific inhibitors [13]. Additionally, it has been demonstrated that STS induced cell death in blood forms of *Trypanosoma brucei* in a dose-dependent manner, stimulating the activation of apoptosis by releasing EndoG from mitochondrial disruption under ROS production [14]; however, to the best of our knowledge there is no information about the antiparasitic effect of streptocarbazole B, 4′-demethylamino-4′-oxostaurosporine, and 7-oxostaurosporine which are the focus of this research. 

Derivative 7-oxostaurosporine (7OSTS) was first isolated from *Streptomyces platensis* [15]. 4′-demethylamino-4′-oxostaurosporine (4′D4′OSTS) from *S. longisporoflavus* [16] and more recently, streptocarbazoles A (SCZ A) and B (SCZ B) from *Streptomyces* sp. FMA strain [17], all of them exerting strong protein kinase inhibitory effects and consequently, acting as anticancer agents. 

**Figure 1:**
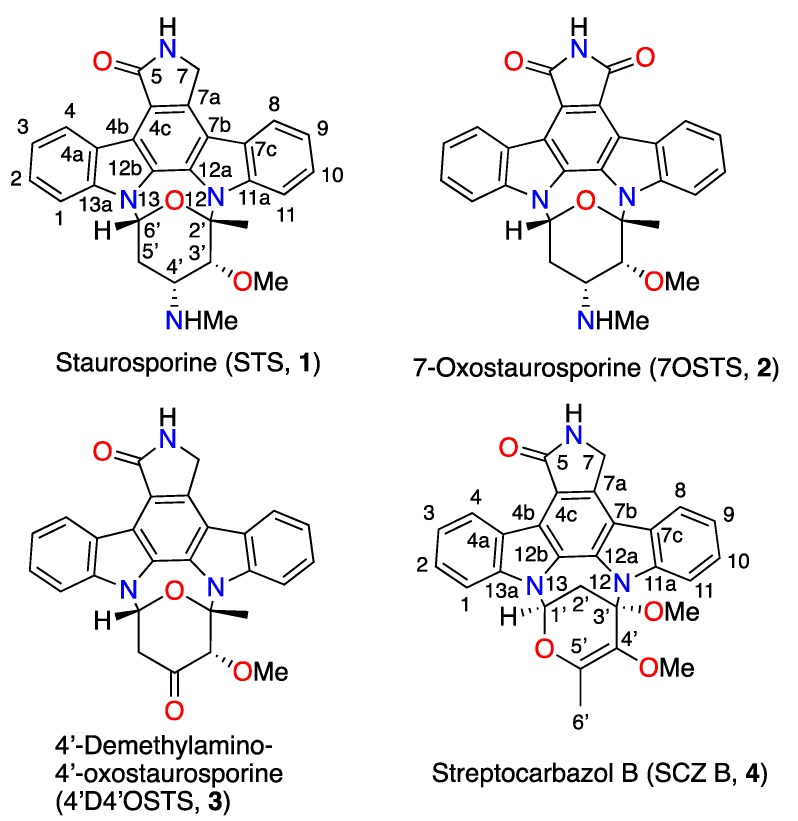
Chemical structures of indolocarbazole (ICZs) metabolites isolated from cultures of *Streptomyces sanyensis* PBLC04 strain.

Infections caused by *Acanthamoeba* spp. affect millions of people worldwide as well as the deficiency of satisfactory treatments. The lack of effectiveness due to drug resistance and high toxicity are the main drawbacks of currently used chemotherapeutics, which evidence that the search for new chemical entities to fight parasitic infections remains a critical need. Recently, we have reported a detailed study on the activity and mechanism of action of STS in *Acanthamoeba* and the programmed cell death via mitochondrial pathway [18]. The aim of this research is based on the isolation and identification of the minor metabolites 7OSTS (**2**), 4′D4′OSTS (**3**), and SCZB (**4**) (Figure 1) from a mangrove-derived bacterium *S. sanyensis* PBLC04 strain collected in the coast of Ecuador, and to value their anti-*Acanthamoeba* activity. Mangroves are singular ecosystems, characterized for their high biodiversity and, hence, ideal for the discovery of new drugs from microbial sources. 

## 2. Results and Discussion

### 2.1. Strain Isolation, Culture, and Identification of Indolocarbazol Metabolites 

*Streptomyces sanyensis* PBLC04 strain was isolated from sediment samples collected in Jambelí mangrove at the southwest coast of Ecuador. 30 L of the strain were cultured using Fernbach bottles and seawater-based modified A1 medium. Cultures of *S. sanyensis* were centrifuged, and the biomass pellet was extracted using a mixture of MeOH:EtOAc:Acetone (2:7:3). Resulting extracts were combined, filtered, and dried in vacuo at 30 °C in a rotary evaporator.

The salt-free biomass extract was chromatographed by filtration on Sephadex-LH-20 with methanol as eluent. The bioassay-guided analysis of the fractions led us to the active fraction SF3 and SF4 against *Acanthamoeba castellanii* Neff with IC_50_ values of 10.68 ± 0.41 µg/mL and 0.44 ± 0.03 µg/mL, respectively. These fractions ware further chromatographed on a RP18 prepacked cartridge applying a gradient elution system of H_2_O:MeOH, buffered with 5 mM NH_4_OAc. The active fractions exhibited characteristic signals for ICZs in the ^1^H-NMR spectrum. Final purification on a silica-gel open column using CHCl_3_:MeOH (9:1) yielded pure staurosporine (**1**) (STS), the major compound, which represents 0.52% of the total content of the biomass extract, whereas pure minor compounds 7-oxostaurosporine (**2**) (7OSTS), 4′-demethylamino-4′-oxostaurosporine (**3**) (4′D4′OSTS), and streptocarbazole B (**4**) (SCZ B) required elution with *n*-Hex:EtOAc:MeOH (2:7:1). The NMR spectra, mass spectrometry, and optical rotation data for compounds **1**–**4** were compared with those previously reported in the chemical literature to confirm their structures [4,15,16,17]. Spectral data and detailed tables with the chemical shifts and HMBC and ROESY correlations are included in the Supplementary Materials.

### 2.2. Anti-Acanthamoeba Activity on Parasite Trophozoites

The antiamoebic activities of natural indolocarbazole metabolites identified in *Streptomyces sanyensis*, compounds **1**–**4**, were tested against trophozoites of the strains: *A. castellanii* Neff, *A. griffini* and *A. polyphaga*. The inhibitory concentrations (IC_50_) were calculated in µM concentrations and are shown in Table 1, and compared with those of chlorhexidine and voriconazole, reference drugs for the treatment of amoebic keratitis (AK), an infection caused by *Acanthamoeba* sp. [19]. Compound 7OSTS (**2**) showed to be as effective as its congener STS (**1**) and the reference compound voriconazole against all tested *Acanthamoeba* spp. Compounds **3** and **4** showed a similar activity and selectivity against the three tested strains, being more active for *A. castellanii* Neff.

Additionally, three commercially available indolocarbazole compounds rebeccamycin (**5**), K252c (**6**), and arcyriaflavin A (**7**) were tested. Compounds **5**–**7** showed to be less active than **1** and **2**, while K252c (**6**) seems to be selective against *A. griffini.*

### 2.3. Cytotoxicity Assays in Murine Macrophages

On the other hand, the potential effectiveness of compounds as antiparasitics depends on their toxicity. As well established in our laboratory [20,21], the toxicity of all compounds was evaluated at the concentration that inhibits 50% of murine macrophages (Table 2). 

Compound **2**, 7OSTS, turned out to be the most cytotoxic with a CC₅₀ of 5.20 ± 1.75 µM. Nevertheless, this CC_50_ value was significantly higher than the IC_50_ values obtained against the strains *A. castellanii* and *A.* graffini. 4′D4′OSTS (**3**) and ICZ B (**4**) were the least toxic among all tested compounds against murine macrophage J774A.1.

### 2.4. In Vitro Activity against Acanthamoeba castellanii Neff Cysts

**Figure 2:**
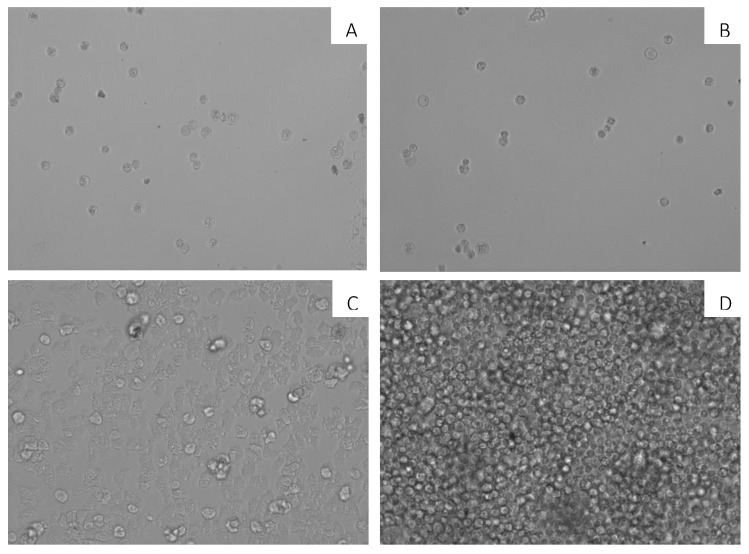
Effect of 7OSTS (**2**) on the cyst of *A. castellanii* Neff at 10 µM (**A**), 2.6 µM (**B**), 0.65 µM (**C**), and negative control (**D**). All images (10×) are representative of the population of treated amoeba, and are based on Live Cell Imaging Microscope EVOS FL Cell Imaging System.

When 7OSTS (**2**) was incubated with mature cysts of *A. castellanii* Neff, non-viable cysts were observed after incubation up to 144 h. In Figure 2, the activity of 7OSTS (**2**) against *A. castellanii* Neff cysts is observed, with an IC_50_ of 0.92 ± 0.33 µM. Non-viable cysts were observed when compared to a negative control of trophozoites and not treated mature cysts. 

### 2.5. Evaluation of the Mechanism of Action of 7OSTS

Among minor compounds, 7OSTS (**2**) was the selected molecule to continue the studies on the mode of action on *A. castellanii* Neff based on the activity values. The search for new therapies which do not produce necrotic cell death is necessary in order to avoid an excessive inflammatory response. Therefore, events involved during programmed cell death or apoptosis-like processes are subject of study and include several morphological stages such as nuclear DNA fragmentation, chromatin condensation or damages at mitochondrial level, among others [22]. Consequently, our next objective was to recognize the mechanism of action of 7OSTS (**2**), through the evaluation of changes in the mitochondrial membrane potential, ATP cell levels, chromatin condensation, and plasmatic membrane integrity. 

**Figure 3:**
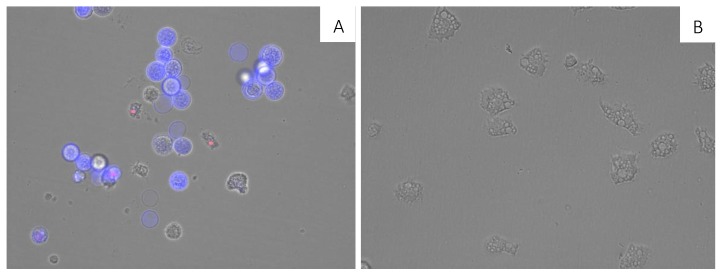
Effect of the IC_90_ concentration of 7OSTS (**2**) on the chromatin condensation at 24 h (**A**) and negative control (**B**) (overlay channels). All images (20×) are representative of the population of treated amoeba and are based on Live Cell Imaging Microscope EVOS FL Cell Imaging System.

A double-stain assay was performed to determine the effect on chromatin condensation. 7OSTS-treated cells at the IC_90_ concentration (2.91 ± 0.67 µM) stained positive in the assay as shown by the bright-blue nuclei stain in Figure 3A, which indicates 7OSTS induces chromatin condensation. Moreover, it is important to highlight that the morphology of the trophozoites treated with IC_90_ of 7OSTS (**2**) (Figure 3A) show rounded and smaller shape compared to the negative control (Figure 3B). which are common events when a cell is under a programmed cell death pathway [22].

**Figure 4:**
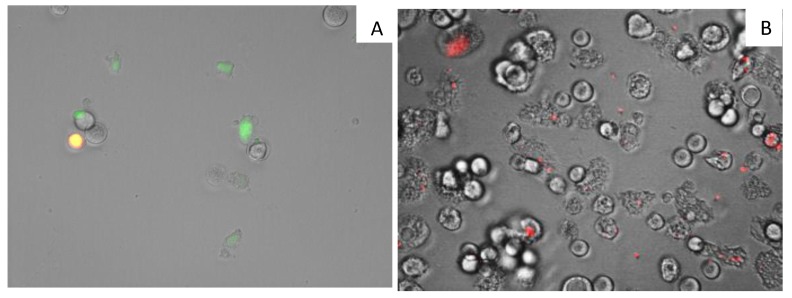
Effect on the mitochondrial potential in amoeba treated with the IC_90_ of 7PSTS for 24 h (**A**) compared with the negative control (**B**) (overlay channels). JC-1 dye remained in the cytoplasm in its monomeric form, green fluorescence, and indicates the collapse of mitochondrial potential (**A**). In negative control (**B**) JC-1 dye accumulates in the mitochondria of healthy cells as aggregates (red fluorescence). All images (20×) are representative of the population of treated amoeba, and are based on Live Cell Imaging Microscope EVOS FL Cell Imaging System.

7OSTS (**2**) also showed an induction of mitochondrial malfunction. Figure 4 shows that *A. castellanii* Neff cells treated with 7OSTS (IC_90_) exhibit higher green fluorescence (**A**) when compared with the negative control (**B**). The green fluorescence indicates the presence of JC-1 (5,5′,6,6′-Tetrachloro-1,1′,3,3′-tetraethyl-imidacarbocyanine iodide) monomers, which reveals a decrease of the mitochondrial membrane potential. In the negative control, the mitochondrial membrane potential is unaltered, showing a red fluorescence caused by JC-1 dimers. 

**Figure 5:**
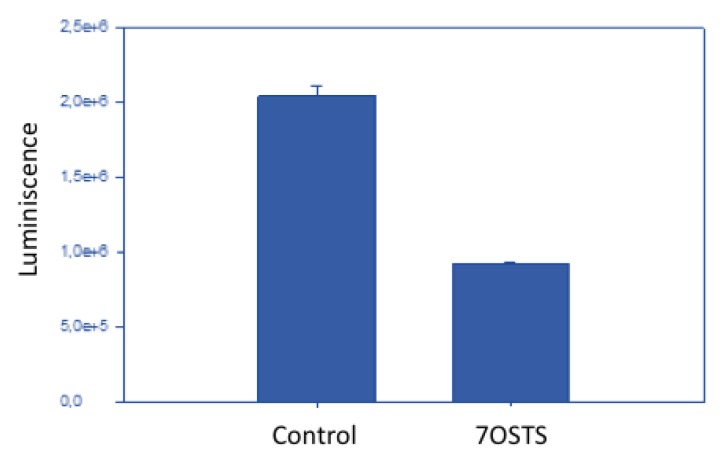
ATP measurement assay with negative control and 7OSTS (**2**).

On the other hand, after incubation of *A. castellanii* Neff with the IC_90_ of 7OSTS (**2**) for 24 h, the ATP level was reduced to 45% compared to the negative control (Figure 5). This result reveals that the cells maintained one half of the ATP level after treatment with 7OSTS, and confirms the mitochondrial damage.

**Figure 6:**
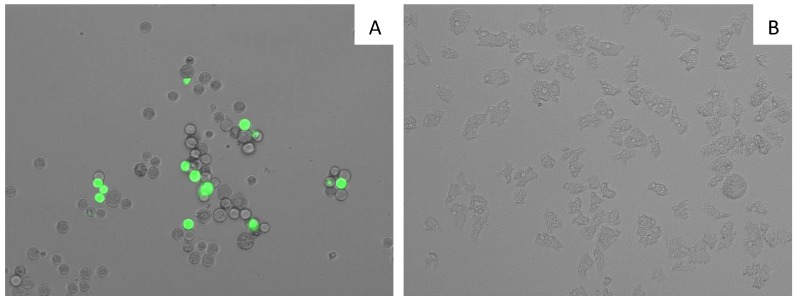
Permeabilization of the *A. castellanii* Neff plasmatic membrane to SYTOX^®^ Green dye caused by addition of IC_90_ of 7OSTS (**2**) (**A**) comparing with the negative control (**B**) (overlay channels). All images (20×) are representative of the population of treated amoeba, and are based on Live Cell Imaging Microscope EVOS FL Cell Imaging System.

7OSTS (**2**) caused plasma membrane permeability in treated cells. Membrane permeabilization experiment on *A. castellanii* Neff cells treated with the IC_90_ of 7OSTS (**2**), showed plasmatic membrane damage after 24 h of treatment. Nevertheless, it is also important to reference that even though the membrane was damaged, cell integrity was maintained as confirmed in Figure 6. 

ICZ derivatives have been the focus of numerous research groups, mainly in the area of chemotherapeutics against cancer, Alzheimer’s disease, and other neurodegenerative disorders [1,2,3]. Thus, several STS analogues have advanced in clinical trials, broadly subdivided into two groups based on their target profile. The first group is represented by STS and includes compounds that are potent inhibitors of protein kinases. On the other hand, the second group are STS analogues modified in the sugar moiety and therefore do not inhibit protein kinases, but instead are used as potent stabilizers of DNA topoisomerase I. Among the natural and biosynthetically produced STS analogues, the 7-hydroxy-derivatives has been tested for clinical phase for several forms of cancer such as pancreas, breast, and lymphoma [23].

The initial studies on the STS-PK complex were directed towards the kinase models PKA and CDK2. These studies and those completed later have indicated the crucial hydrogen bond interactions between the heteroatoms of the lactam moiety of the alkaloids with a conserved glutamic acid residue (Glu). On the other side, the methyl amino group at C-4′ of the sugar moiety is involved in the formation of two hydrogen bonds with amino acid residues such as Glu and aspartic acid (Asp), part of the protein catalytic site. These interactions force a boat conformation of the sugar ring of the ligand and a perpendicular orientation to the planar aromatic indolocarbazole nucleus when the ICZ-family of compounds inhibit protein kinases. Another important point is that the binding affinity of ICZs increases with the presence of hydrogen bonds between the nitrogen of the methyl amino moiety at C-4′ with neighboring protein residues [6,24].

**Figure 7:**
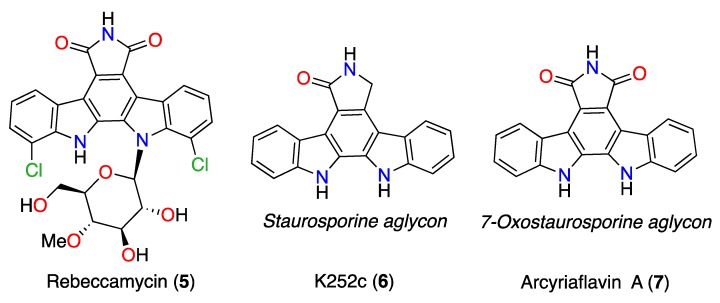
Commercial ICZs: rebeccamycin (**5**), K252c (**6**), and arcyriaflavin A (**7**).

Commercial ICZ compounds obtained from their natural sources are available for biochemical studies (Figure 7). Rebeccamycin (**5**) is an indolocarbazole antibiotic produced, among other actinomycetes, by *Saccharothrix aerocolonigenes* [25]. It is a DNA-binding molecule that inhibits topoisomerase I activity [26,27,28,29]. Rebeccamycin shows cytotoxic activity against human lung, colon, and nasopharyngeal carcinoma cell lines with IC_50_ values that range from 0.4–98 µM [30]. The STS aglycon is known as K252c (**6**). It is an efficient protein kinase C (PKC) inhibitor (IC_50_ 2.45 µM) compared to its inhibitory protein kinase A (PKA) activity (IC_50_ 25.7 µM) [26]. Arcyriaflavin A (**7**), the 7OSTS aglycon, is an inhibitor of cyclin-dependent kinase 4 (CDK4) with an IC_50_ = 140 nM, and calcium/calmodulin-dependent protein kinase II (CaMKII; IC_50_ = 25 nM), showing selectivity for CDK4 and CaMKII over protein kinase A (PKA; IC_50_ > 2 µM) and PKC (IC_50_ >100 µM) [2,31].

**Figure 8:**
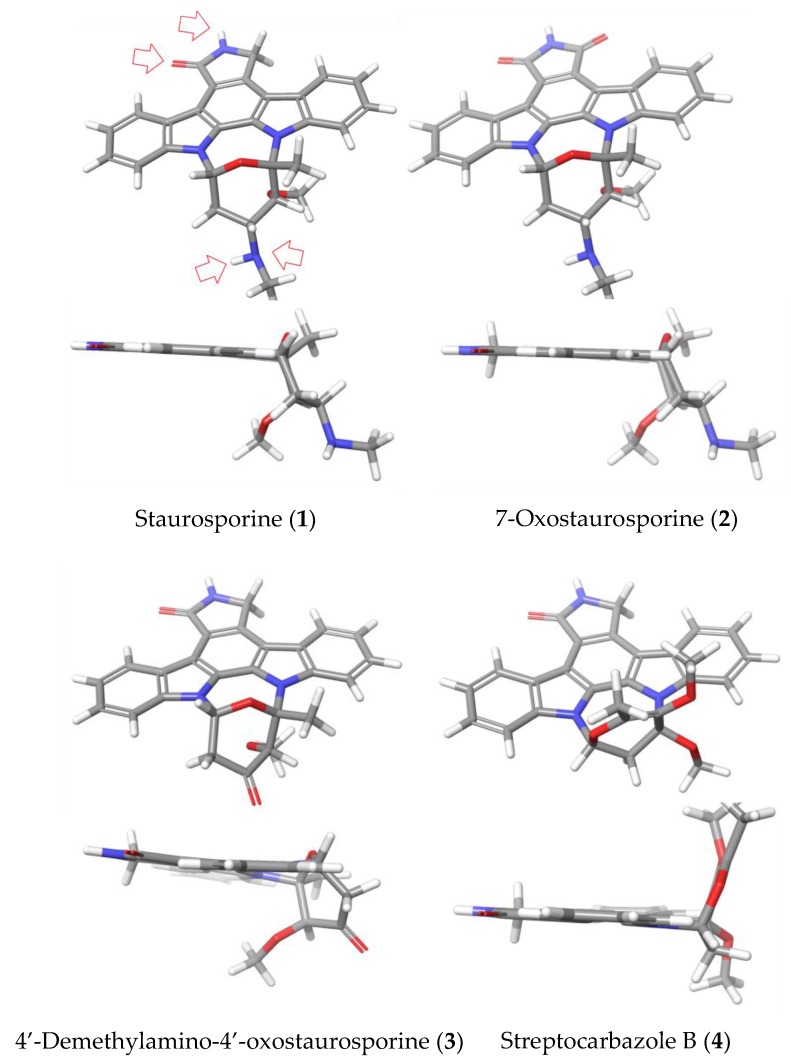
3D Chemical structures for (ICZs) metabolites isolated from *Streptomyces sanyensis* cultures. Top and perpendicular views to the indol system. Red arrows indicate the interaction sites of stauroporine (STS, **1**) with protein kinase type CDK2 models. Minimized structures were obtained using Maestro, version 10.2, Schrödinger, LLC, New York, NY, 2015.

In this work, we have found a clear correlation between the antiparasitic activities observed and the structural elements and conformations of the indolocarbazole molecules under study (Figure 8). Thus, both STS (**1**) and the 7-oxo derivative (**2**) showed potent activities against the tested species. This fact could be explained based on the conserved interactions reported for many protein kinases, which establish interactions between the lactam group and methyl amine at the C-4′ position of both compounds. This result agrees with a similar orientation and conformation of **1** and **2**. In addition, in the particular case of *A. polyphaga*, a difference of activity between STS (**1**) and 7OSTS (**2**) was observed, that could be justified based on the positive or negative interaction with the active site of the target PKs, due to the additional functionalization at C-7 position of **2**.

On the other hand, compounds 4′D4′OSTS (**3**) and SCZ B (**4**) showed moderate antiamoebic activity compared to **1** and **2**. In the case of **3**, it might be consequence of the absence of the expected hydrogen bond with the methyl amine group at C-4′ in the PKs inhibition mechanism by ICZs (Figure 8). For compound **4**, a significant structural change in the sugar ring is observed which eliminate all these conserved interactions at C-4′ position. Furthermore, the different orientation of the sugar moiety could produce strong steric impediments at the active site. Similarly, this structural feature seems to be the cause of the reduced toxicity of **3** and **4** (CC_50_ > 40 μM), which confer these compounds a similar selective index to the active 7OSTS (**2**) in treatments against the *A. castellanii* Neff strain. The results obtained with commercial ICZs (**5**–**7**) reinforce the relevance of the interactions due to the sugar moiety of STS (**1**) and the 7OSTS (**2**) to improve the antiamoeboid activity.

## 3. Materials and Methods

### 3.1. General Experimental Procedures

NMR spectra were recorded on a Bruker AVANCE 500 MHz or 600 MHz (Bruker Biospin, Falländen, Switzerland), as required. NMR spectra were obtained dissolving samples in CDCl_3_ (99.9%) and chemical shifts are reported relative to solvent (δ_H_ 7.26 and δ_C_ 77.0 ppm). Bruker AVANCE 600 MHz instrument is equipped with a 5 mm TCI inverse detection cryoprobe (Bruker Biospin, Falländen, Switzerland). Standard Bruker NMR pulse sequences were utilized. Optical rotations were measured in CHCl_3_ on a PerkinElmer 241 polarimeter (Waltham, MA, USA) by using a Na lamp. HR-ESI-MS data were obtained on a Waters LCT Premier XE Micromass (Manchester, UK) and VG-AutoSpec Micromass spectrometers (Manchester, UK), respectively. IR spectra were recorded on a Bruker IFS66/S (Ettlingen, Germany) equipped with an ATR accessory using CHCl_3_ solutions. EnSpire ^®^ Multimode Reader (Perkin Elmer, Waltham, MA, USA) using absorbance values of alamarBlue^®^ reagent (Bio-Rad Laboratories, Oxford, UK). HPLC (high performance liquid chromatography) separations were carried out with an Agilent 1260 Infinity Quaternary LC equipped with a Diode Array Detector (Waldbronn, Germany). TLC (Thin layer chromatography) (Merck, Darmstadt, Germany) was visualized by UV light (254 nm) and spraying with cobalt chloride reagent (2% in sulfuric acid, 10%) and heating.

### 3.2. Biological Material

*Streptomyces sanyensis* PBLC04 strain was collected in Jambelí mangrove (3°15′792″ S, 80°00′739″ W–03°17′711″ S, 80°01′924″ W), Ecuador. The strain is included in the microbial collection of Universidad Técnica Particular de Loja (UTPL, Loja-Ecuador). Samples of marine sediment were collected in 15 mL Falcon tubes from 1 to 3 m depth and put into sterile polythene bags. Stamps of pulverized samples were seeded on agar plates of a sea water-based medium (10 g starch, 4 g yeast extract, 2 g peptone, 1 g CaCO _3_, 40 mg Fe_2_(SO _4_)_3_ 4H_2_O, 100 mg KB, 15 g Bacto Agar, per liter), acidic gauze medium, humic acid medium, and TCG medium, all of them containing 1% of polymyxin B and cycloheximide as inhibitors of undesired growth. The plates were incubated at 30 °C and observed periodically. Gram staining was used to identify branched filamentous Gram-positive bacteria.

### 3.3. Cultures and Preparation of Crude Extracts

*Streptomyces sanyensis* PBLC04 strain was cultured in Fernbach bottles using a modified seawater-based medium (A1) that consisting of 75% seawater containing 10 g starch, 4 g yeast extract, 2 g proteose peptone, 1 g calcium carbonate, supplemented with 5 mL/L of a solution of potassium bromide (67 mM) and ferric sulfate (20 mM). Fernbach flasks with 1 L each were maintained in an orbital shaker at 30 °C and 200 rpm for 7 days. Cultures of *S. sanyensis* PBLC04 were then centrifuged at 5000 rpm for 10 min at 4 °C to collect the cell biomass. Supernatant was treated by Amberlite^®^ XAD7-HP resin (20 g/L) addition and stirred for 3 h for the recuperation of excreted substances. After filtration, both the resin and the biomass pellet were separately extracted using a mixture of MeOH:EtOAc:Acetone (2:7:3) for 12 h at 120 rpm in an orbital shaker. Resulting extracts were filtered, dried in vacuo at 30 °C in a rotary evaporator and stored a −80 °C until used.

### 3.4. Isolation of Indolocarbazole Metabolites

The obtained crude extract 12.6 g was fractionated by gel filtration on Sephadex LH-20 column, eluting with methanol to yield 10 fractions that were finally gathered in four final fractions according to TLC and ^1^H-NMR analysis. Thin layer chromatography (TLC) monitoring was used with cobalt chloride (2%) as spraying reagent. The bioassay-guided analysis of the Sephadex LH-20 fractions led us to an active fraction SF3 and SF4 against *Acanthamoeba castellanii* Neff with IC_50_ values of 10.68 ± 0.41 µg/mL and 0.44 ± 0.03 µg/mL, respectively; and against *Leishmania amazonensis* with IC_50_ values of 0.43 ± 0.07 µg/mL and 0.08 ± 0.01 µg/mL. These fractions were then chromatographed using a flash chromatography on a RP18 prepacked cartridge (25–40 µm, 70 g, Götec-Labortechnik GmbH) operating in a gradient elution protocol of H_2_O:MeOH, 5mM NH_4_OAc, (20% to 100% MeOH) at 2 mL/min flow. All process was monitored by UV detection at 254 nm. The active fractions those exhibited characteristic signals for ICZs in the ^1^H-NMR spectrum and bioactivities against cited parasites were separated by elution on Si-60 open column (230–400 mesh, 60 Å) using CHCl_3_:MeOH (9:1) yielding 65.9 mg of pure staurosporine (**1**) (STS, 65.6 mg, 0.521%) from SF3; and *n*-Hex:EtOAc:MeOH (2:7:1) to obtained pure the compounds 7-oxostaurosporine (**2**) (1.01 mg, 0.008%), 4′-demethylamino-4-oxostaurosporine (**3**), (1.13 mg, 0.009%) and streptocarbazole B (**4**) (SCZ B, 0.91 mg, 0.007%) from SF4.

#### 3.4.1. Staurosporine, (STS), (1)

Yellow powder, [α]${}_{D}^{20}$ + 43 (*c* 0.3, CHCl_3_); UV (CHCl_3_) λ_max_ (log ε) 297 (4.48) nm. IR υ_max_ 2920, 2851, 1713, 1568, 1463, and 1318 cm^−1^. HRESIMS *m/z* 489.1908 [M + Na]^+^, calc. 489.1897 for C_28_H_24_N_4_O_3_Na. ^1^H and ^13^C NMR data see Table S1. ^1^H and ^13^C NMR spectra, see Supplementary Materials (Figures S2 and S3).

#### 3.4.2. 7-Oxostaurosporine, (7OSTS), (2)

Yellow powder, [α]${}_{D}^{20}$ +32.0° (*c* 0.1, CHCl_3_); UV (CHCl_3_) λ_max_ (log ε) 243, 265, 288, 305, 319 nm (ε 319 nm: 20513.39 L mol^−1^ cm^−1^) nm. IR υ_max_ 2920, 2851, 1713, 1568, 1463, and 1318 cm^−1^. HRESIMS *m/z* 481.1875 [M + H]^+^ calcd. 481.1876 for C_28_H_25_N_4_O_4_. ^1^H and ^13^C NMR data see Table S2. ^1^H and ^13^C NMR spectra, see Supplementary Materials (Figures S5 and S6).

#### 3.4.3. 4’-Demethylamino-4′-oxostaurosporine, (4′D4′OSTS), (3)

Yellow powder, [α]${}_{D}^{20}$ 22° (*c* 0.12, CHCl_3_); UV (CHCl_3_) λ_max_ (log ε) 248, 268, 293, 319, 335, 352, 368 nm (ε_293nm_ 23106.25 cm^−1^M^−1^) nm. IR υ_max_ 2922, 2853, 2362, 1682., 1456 and 1317 cm^−1^. HRESIMS *m/z* 474.1425 [M + Na]^+^ calcd. 474.1430 for C_27_H_21_N_3_O_4_Na. ^1^H and ^13^C NMR data, see Table S3. ^1^H and ^13^C NMR spectra, see Supplementary Materials (Figures S8 and S9).

#### 3.4.4. Streptocarbazole B, (SCZ B), (4)

Pale yellow powder, [α]${}_{D}^{20}$ −27° (*c* 0.09, CHCl_3_); UV (CHCl_3_) λ_max_ (log ε) Its UV spectrum show bands at 245, 268, 291, 318, 334, 348, 366 nm (ε 291nm: 22458.82 cm^−1^M^−1^) nm. IR υ_max_ 2928, 1682, and 1456 cm^−1^. HRESIMS *m/z* 488.1513 [M + Na]^+^ calcd. 488.1586 for C_28_H_23_N_3_O_4_Na. ^1^H and ^13^C NMR data see Table S4. ^1^H and ^13^C NMR spectra, see Supplementary Materials (Figures S11 and S12).

### 3.5. Commercial ICZ Analogs ***5**–**7***

Indolocarbazole derivatives rebeccamycin (**5**) (CAS no. 93908-02-2), K252c (**6**) (CAS no. 85753-43-1), and arcyriaflavin A (**7**) (CAS no. 118458-54-1), were all acquired from Cayman Chemical (Ann Arbor, MI, USA).

### 3.6. Cell Culture

The amoeba strain used in this study was the type strain: *Acanthamoeba castellanii* Neff (ATCC 30010) which was axenically grown in PYG medium (0.75% (*w*/*v*) proteose peptone, 0.75% (*w*/*v*) yeast extract and 1.5% (*w*/*v*) glucose) containing 40 µg/mL of gentamicin (Biochrom AG, Cultek, Granollers, Barcelona, Spain). The murine macrophages J774A.1 (ATCC TIB-67) cell line was cultured in RPMI 1640 medium supplemented with 10% fetal bovine serum at 37 °C and 5% CO_2_ atmosphere, was used for the cytotoxicity assays. Successively, the evaluated molecules were also tested against two clinical isolates, *Acanthamoeba* *griffini*, genotype T3 obtained in a previous study [32], and *Acanthamoeba polyphaga* genotype T4 ATCC 30461, both axenically grown in the same medium as *A*. *castellanii* Neff.

### 3.7. In Vitro Activity Against Acanthamoeba spp. Trophozoites

To evaluate the biological activity of the fractions and molecules, the anti-*Acanthamoeba* experiments were carried out using the alamarBlue^®^ colorimetric assay, as it was previously described [22]. Briefly, *Acanthamoeba* strain was seeded in triplicate on 96-well microtiter plates (ThermoFisher™, Waltham, MA, USA) with 50 µL from a stock solution of 5 × 10^4^ cells/mL and 50 µL of serial dilutions of the evaluated molecules in each well (1% DMSO was used to dissolve the highest dose of the compounds with no effects on the parasites). Two reference treatments [33] such as chlorhexidine (chlorhexidine digluconate; Alfa Aesar) and voriconazole (Sigma), were used as positive controls. Finally, the alamarBlue^®^ Reagent (Life Technologies, Madrid, Spain) was placed into each well at an amount equal to 10% of the final volume. Plates were incubated for 96 h at 26 °C with a slight agitation until their measurement in the EnSpire^®^ Multimode Plate Reader (Perkin Elmer, Madrid, Spain) using fluorescence, at 570/585 nm.

### 3.8. In Vitro Activity against Acanthamoeba Castellanii Neff Cysts

On the other side, cysticidal activity of 7OSTS (**2**) was determined also using the alamarBlue™ reagent and confirmed visually by inverted microscopy. Cysts of *A. castellaniii* Neff were prepared as previously described [34,35]. First of all, trophozoites were transferred from PYG medium based cultures (trophozoite medium) to Neff’s encystment medium (NEM; 0.1 M KCl, 8 mM MgSO4·7H_2_O, 0.4 mM CaCl2·2H_2_O, 1 mM NaHCO_3_, 20 mM ammediol [2-amino-2-methyl-1,3-propanediol; Sigma Aldrich Chemistry Ltd., Madrid, Spain], pH 8.8, at 25 °C) and were cultured in this medium with gently shaking for a week in order to obtain mature cysts. In order to carry out the cysticidal assay, mature cysts were harvested and washed twice with PYG medium. Serial dilutions of 7OSTS (**2**) were made in PYG and place onto sterile 96-well microtiter plates. 5·10^4^ mature cysts of *Acanthamoeba*/mL were added to the wells with 50 µL of 7OSTS (**2**) dilutions. After 7 days of incubation, the plate was centrifuged at 3000 rpm for 10 min. The supernatant was removed and replaced with 100 µL of fresh PYG medium in each well. Finally, 10 μL of the alamarBlue^®^ Reagent was placed into each well. The plates were then incubated for 144 h at 28 °C and the emitted fluorescence was measured with the EnSpire^®^ Multimode Plate Reader (Perkin Elmer, Madrid, Spain) using fluorescence, at 570/585 nm.

### 3.9. Cytotoxicity Test

The cytotoxicity effect of active molecules was evaluated in murine macrophage J774A.1 cell line (ATCC # TIB-67) following the same protocol described above for *Acanthamoeba* trophozoites. Then, plates containing alamarBlue^®^ were incubated for 24 h at 37 °C in presence of CO_2_ at 5%.

### 3.10. Statistical Analysis

The percentage of the growth inhibition, 50% inhibitory concentration (IC_50_ or CC_50_), was calculated by nonlinear regression analysis with 95% confidence limits using Sigma Plot 12.0 statistical analysis software (Systat Software). All experiments were performed three times, and the mean values were also calculated. A paired two-tailed *t*-test was used for analysis of the data. Values of *p* < 0.05 were considered significant.

### 3.11. Double-Stain Assay for Programmed Cell Death Determination

10^5^ amoebas/well were incubated in a 24-well plate with the previously calculated IC_90_ of 7OSTS for 24 h. A double-stain apoptosis detection kit (Hoechst 33342/PI) (ThermoFisher™, Waltham, MA, USA) was used to carry out the experiment following the manufacturer’s recommendations. The images were obtained using the EVOS FL Cell Imaging System AMF4300, Life Technologies, USA. The double-staining pattern allows the identification of live cells with low level of fluorescence, cells undergoing PCD with a higher level of blue fluorescence (as chromatin condenses), and dead cells which will show low-blue and high-red fluorescence (as the propidium iodide stain enters the nucleus of dead cells).

### 3.12. Analysis of Mitochondrial Membrane Potential

The use of JC-1 mitochondrial membrane potential detection kit (Cayman Chemical) it is possible to detect the collapse of an electrochemical gradient across the mitochondrial membrane during apoptosis process. 10^5^ amoebas/mL were treated with IC_90_ of 7OSTS (**2**) for 24 h, the cells were centrifuged (1000 rpm × 10 min) and suspended in JC-1 buffer. Images were taken on the EVOS FL inverted microscope. The staining pattern allows the identification of two groups in a cellular population: live cells will show only red fluorescence, but cells with low mitochondrial potential (undergoing PCD) will show a higher level of green fluorescence.

### 3.13. Measurement of ATP Levels

An appropriate level of ATP is necessary to maintain the cell metabolism. The effect of the drug on the ATP production was evaluated by incubating (10^5^) of cells/mL with the previously calculated IC_90_ of 7OSTS (**2**) using the CellTiter-Glo Luminescent Cell Viability Assay. The luminescence of each assay was measured on the EnSpire^®^.

### 3.14. Plasma Membrane Permeability 

In order to detect alterations of the membrane permeability in treated cells, the SYTOX Green (ThermoFischer^TM^) assay was performed. Briefly, 10^5^ trophozoite were washed and incubated in saline solution with SYTOX Green at a final concentration of 1 μM for 15 min in darkness. After that, IC_90_ of 7OSTS (**2**) was added to the cells and incubated for 24 h. Cells were observed and pictures were taken on the EVOS FL inverted microscope.

## 4. Conclusions

It is known that infections caused by *Acanthamoeba* spp. affect millions of people worldwide as well as the deficiency of satisfactory treatments. The lack of effectiveness due to drug resistance, and high toxicity are the main drawbacks of currently used drugs. Current therapeutic options against AK rely on the application of biguanides (mainly chlorhexidine or polyhexamethylene biguanide (PHMB)) and azoles (voriconazole) both as eye drops and/or topical. These agents are able to eliminate the trophozoite stage, however the concentrations needed to kill the cysts are usually higher and thus toxic to the eye of the patients. Moreover, these options are lengthy and require application even each hour complicating patients’ healing [28]. Therefore, there is an urgent need to develop new agents to treat AK.

Based on the knowledge of indolocarbazole-type metabolites and their interactions with protein kinases, in this work we have been able to hypothesize about their response against *Acanthamoeba* parasites. Thus, assuming STS (**1**) as a leader compound, it is possible to conclude that in *Acanthamoeba* spp., both **1** and **2**, seem to present similar interactions with the parasite PKs and, therefore, both compounds show similar ranges of inhibition. Similar structural analysis allowed to predict that, for compounds **3** and **4**, minor activities should be expected. Among minor metabolites, 7OSTS (**2**) was the most active. The study of its mode of action on *A. castellanii* Neff trophozoites showed that 7OSTS (**2**) induced chromatin condensation and triggered important morphological changes of cells, affecting membrane permeability, and causing mitochondrial damage. 

This study reveals the ICZ family as potential molecules or biological tools to explore the mechanism of action and the structure of other families of protein kinases. At the moment, there are not known drugs targeting protein kinases of *Acanthamoeba* spp., fact that increases the value of ICZ compounds. Protein kinases could be an alternative and relevant target to fight against amoeboid infections.

## Figures and Tables

**Table 1:** Effects of ICZ metabolites isolated from *Streptomyces sanyensis* against *Acanthamoeba* species. IC_50_ are reported in µM concentrations (Mean concentration ± SD). * Reference compounds.

Compounds	*A. castellanii* Neff	*A. griffini*	*A. polyphaga*
STS (**1**) [18]	0.57 ± 0.12	0.97± 0.12	2.25 ± 0.42
7OSTS (**2**)	0.83 ± 0.09	0.96 ± 0.19	5.51 ± 0.09
4’D4’OSTS (**3**)	7.53 ± 0.73	23.86 ± 0.55	>40
ICZ B (**4**)	6.83 ± 2.60	14.51 ±0.38	>40
Rebeccamycin (**5**)	5.37 ± 0.23	5.02 ± 0.18	7.47 ± 0.61
K252c (**6**)	6.17 ± 0.73	2.25 ± 0.32	5.59 ± 0.34
Arcyriaflavin A (**7**)	7.32 ± 0.45	7.53 ± 1.11	7.59 ± 0.73
Chlorhexidine *	3.02 ± 0.89	3.73 ± 0.98	4.76 ± 0.08
Voriconazole *	0.94 ± 0.29	0.10 ± 0.02	10.10 ± 2.21

**Table 2:** Toxicity of compounds **1**–**4** against murine macrophage J774A.1 (CC_50_) measured by alamarBlue^®^ assay; and CC_50_ are reported in µM concentrations (Mean concentration ± SD). *Reference compounds.

Compounds	Macrophage J774A.1 CC_50_ (µM)
STS (**1**)	8.74 ± 0.72
7OSTS (**2**)	5.20 ± 1.75
4’D4’OSTS (**3**)	>40
ICZ B (**4**)	>40
Chlorhexidine *	7.40 ± 0.39
Voriconazole *	7.56 ± 0.77

## Supplementary Materials

The following are available online at https://www.mdpi.com/1660-3397/17/10/588/s1, Scheme S1: Isolation and purification of metabolites from *Streptomyces sanyensis*; Figure S1: Chemical structure, atom carbon position numbers and physical data for staurosporine (STS) **1**; Table S1: NMR data for staurosporine (STS) **1** in CD_2_Cl_2_ (600 MHz, 298 K), Figure S2: ^1^H NMR spectrum of staurosporine (**1**) in CD_2_Cl_2_ (600 MHz, 298 K); Figure S3: ^13^C NMR spectrum of staurosporine (**1**) in CD_2_Cl_2_ (150 MHz, 298 K); Figure S4: Chemical structure, atom carbon position numbers and physical data for 7-oxostaurosporine (**2**); Table S2: NMR data for 7-oxostaurosporine (**2**) in CD_2_Cl_2_ (600 MHz, 298 K); Figure S5: ^1^H NMR spectrum of 7-oxostaurosporine (**2**) in CD_2_Cl_2_ (600 MHz, 298 K); Figure S6: ^13^C NMR spectrum of 7-oxostaurosporine (**2**) in CD_2_Cl_2_ (150 MHz, 298 K); Figure S7: Chemical structure, atom carbon position numbers and physical data for 4′-demethylamino-4′-oxostaurosporine (**3**); Table S3: NMR data for 4′-demethylamino-4′-oxostaurosporine (**3**) in CD_2_Cl_2_ (600 MHz, 298 K); Figure S8: ^1^H NMR spectrum of 4′-demethylamino-4′-oxostaurosporine (**3**) in CD_2_Cl_2_ (600 MHz, 298 K); Figure S9: ^13^C NMR spectrum of 4′-demethylamino-4′-oxostaurosporine (**3**) in CD_2_Cl_2_ (150 MHz, 298 K); Figure S10: Chemical structure, atom carbon position numbers and physical data for streptocarbazole B (SCZ B, **4**); Table S4: NMR data for streptocarbazole B (SCZ B, **4**) in CD_2_Cl_2_ (600 MHz, 298 K); Figure S11: ^1^H NMR spectrum of streptocarbazole B (SCZ B, **4**) in CD_2_Cl_2_ (600 MHz, 298 K); Figure S12: ^13^C NMR spectrum of streptocarbazole B (SCZ B, **4**) in CD_2_Cl_2_ (150 MHz, 298 K).
