# Supplementary material for: Antiamoebic Activities of Indolocarbazole Metabolites Isolated from Streptomyces sanyensis Cultures
Source: Mar Drugs. 2019 Oct 17;17(10):588. doi: 10.3390/md17100588 (PMC6836125; doi:10.3390/md17100588)

Supplementary Material

Antiamoebic activities of indolocarbazole metabolites isolated from *Streptomyces sanyensis* cultures

Luis Cartuche[1,2], María Reyes-Batlle[3], Ines Sifaoui[3], Iñigo Arberas-Jiménez[3], José E. Piñero[3], José J. Fernández[1,4], Jacob Lorenzo-Morales[3]* and Ana R. Díaz-Marrero[1]*

[1] Instituto Universitario de Bio-Orgánica Antonio González (IUBO AG), Centro de Investigaciones Biomédicas de Canarias (CIBICAN), Universidad de La Laguna (ULL), Avda. Astrofísico F. Sánchez 2, 38206 La Laguna, Tenerife, Spain; jjfercas@ull.edu.es (J.J.F.)

[2] Departamento de Química y Ciencias Exactas, Sección Química Básica y Aplicada, Universidad Técnica Particular de Loja (UTPL), San Cayetano alto s/n, A.P. 1101608, Loja, Ecuador; lecartuche@utpl.edu.ec (L.C.)

[3] Instituto Universitario de Enfermedades Tropicales y Salud Pública de Islas Canarias, Universidad de La Laguna, Avda. Astrofísico F. Sánchez s/n, 38206 La Laguna, Tenerife, Spain; mreyesba@ull.edu.es (M.R.-B.); isifaoui@ull.edu.es (I.S.); alu0101283227@ull.edu.es (I.A.J.); jpinero@ull.edu.es (J.E.P.)

[4] Departamento de Química Orgánica, Universidad de La Laguna (ULL), Avda. Astrofísico F. Sánchez, 2, 38206 La Laguna, Tenerife, Spain

Table of Contents

	Contents	Page
1	**Scheme 1.** Isolation and purification of metabolites from *Streptomyces sanyensis*	S3
2	**Figure S1**. Chemical structure, atom carbon position numbers and physical data for staurosporine (STS) **1**	S4
3	**Table S1**. NMR data for staurosporine (STS) **1** in CD_2Cl_2 (600 MHz, 298 K)	S5
4	**Figure S2.** ^{1}H NMR spectrum of staurosporine (**1**) in CD_2Cl_2 (600 MHz, 298 K)	S6
5	**Figure S3.** ^{13}C NMR spectrum of staurosporine (**1**) in CD_2Cl_2 (150 MHz, 298 K)	S7
6	**Figure S4**. Chemical structure, atom carbon position numbers and physical data for 7-oxostaurosporine (**2**).	S8
7	**Table S2**. NMR data for 7-oxostaurosporine (**2**) in CD_2Cl_2 (600 MHz, 298 K)	S9
8	**Figure S5.** ^{1}H NMR spectrum of 7-oxostaurosporine (**2**) in CD_2Cl_2 (600 MHz, 298 K)	S10
9	**Figure S6.** ^{13}C NMR spectrum of 7-oxostaurosporine (**2**) in CD_2Cl_2 (150 MHz, 298 K)	S11
10	**Figure S7**. Chemical structure, atom carbon position numbers and physical data for 4'-demethylamino-4'-oxostaurosporine (**3**).	S12
11	**Table S3**. NMR data for 4'-demethylamino-4'-oxostaurosporine (**3**) in CD_2Cl_2 (600 MHz, 298 K)	S13
12	**Figure S8.** ^{1}H NMR spectrum of 4'-demethylamino-4'-oxostaurosporine (**3**) in CD_2Cl_2 (600 MHz, 298 K)	S14
13	**Figure S9.** ^{13}C NMR spectrum of 4'-demethylamino-4'-oxostaurosporine (**3**) in CD_2Cl_2 (150 MHz, 298 K)	S15
14	**Figure S10**. Chemical structure, atom carbon position numbers and physical data for streptocarbazole B (SCZ B, **4**)	S16
15	**Table S4**. NMR data for streptocarbazole B (SCZ B, **4**) in CD_2Cl_2 (600 MHz, 298 K)	S17
16	**Figure S11.** ^{1}H NMR spectrum of streptocarbazole B (SCZ B, **4**) in CD_2Cl_2 (600 MHz, 298 K)	S18
17	**Figure S12.** ^{13}C NMR spectrum of streptocarbazole B (SCZ B, **4**) in CD_2Cl_2 (150 MHz, 298 K)	S19

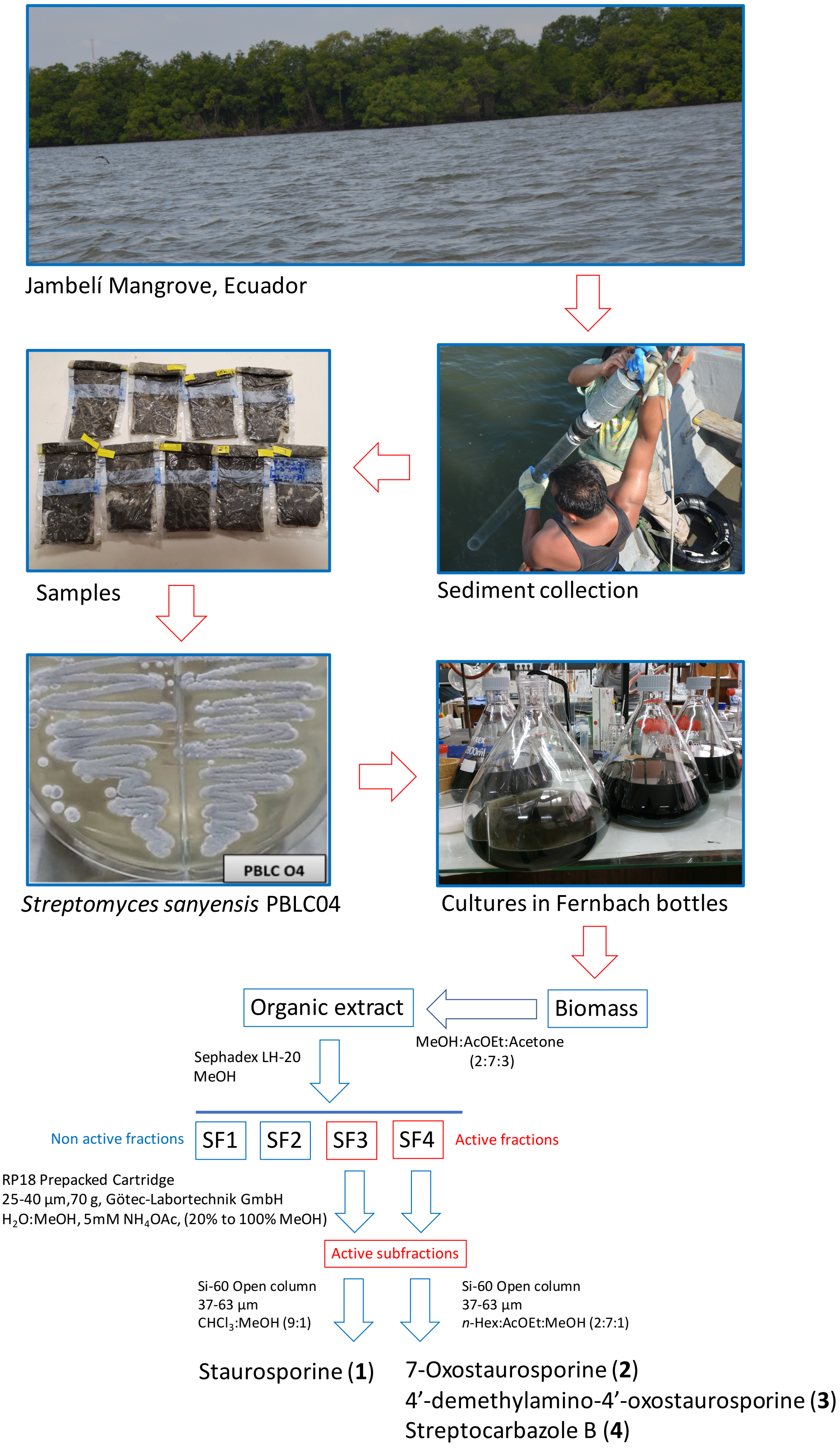

Scheme 1. Samples collection, isolation, culture and purification procedure for metabolites from *Streptomyces sanyensis*.

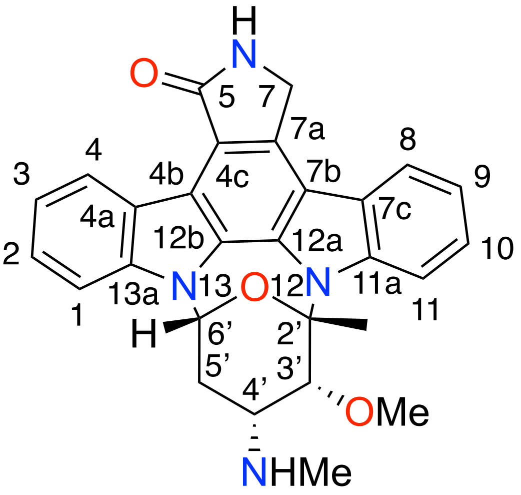

Staurosporine (STS, **1**)

Yellow powder, $[\alpha]_D^{20}$ + 43 (*c* 0.3, $CHCl_3$)
UV ($CHCl_3$) λ_{max} (log ε) 297 (4.48) nm
IR υ_{max} 2920, 2851, 1713, 1568, 1463 and 1318 cm^{-1}
HRESIMS *m/z* 489.1908 $[M+Na]^+$, calc. 489.1897 for $C_{28}H_{24}N_4O_3Na$

Figure S1. Chemical structure, atom carbon position numbers and physical data for staurosporine (STS, **1**).

Table S1. NMR data for staurosporine (STS) **1** in CD_2Cl_2 (600 MHz, 298 K)

Carbon	δ^{1}H	δ ^{13}C	HMBC	ROESY
**1**	7.35, d, *J*=8.1 Hz, 1H)	107.8	4a	
**2**	7.49 (t, *J* = 7.8 Hz, 1H)	125.5	13a	
**3**	7.34 (t, *J*, 7.8 Hz, 1H)	120.0	1	
**4**	9.36 (d, *J* = 7.6 Hz, 1H)	126.7	2, 4b, 13a	
**4a**	-	123.8		
**4b**	-	115.5		
**4c**	-	118.9		
**5**	-	174.0		
**6**	6.73 (s, 1H) NH	-	5, 7a, 4c	
**7**	5.00 (dd, *J*= 1.7, 1.0 Hz, 2H)	46.5	5, 7a, 7b, 4c, 12ª (J^4 weak)	
**7a**	-	133.0		
**7b**	-	114.6		
**7c**	-	125.0		
**8**	7,90 (d, J = 7.8 Hz, 1H)	121.1		7
**9**	7.30 (t, *J* = 7.5 Hz, 1H)	120.4		
**10**	7.42 (t, *J* = 8.1 Hz, 1H)	124.7	11a	
**11**	7.96 (d, J = 8.5 Hz, 1H)	116.0	7c, 9	2'-CH_3
**11a**	-	140.5		
**12b**	-	127.7		
**12a**	-	131.3		
**13a**	-	137.2		
**2'**	-	91.6		
**3'**	3.89 (dd, *J* = 3.8, 1.2 Hz, 1H)	84.6	5', 3'-OCH_3	2'-CH_3, 5'α, 5'β
**4'**	3.37(q, *J* = 3.8 Hz, 1H)	50.7		4'-NCH_3
**5'α**	2.44 (ddd, *J* = 14.9, 5.8, 3.8 Hz, 1H)	30.6		
**5'β**	2.74 (ddd, *J* = 14.8, 4.2, 1.5 Hz, 1H)	30.6		
**6'**	6.56 (dd, *J* = 5.6, 1.2 Hz, 1H)	80.7	2', 4', 12b	1
**2'-CH_3**	2.35 (s, 3H)	30.5	2', 3'	
**3'-OCH_3**	3.44 (s, 3H)	57.6	3'	
**4'-NCH_3**	1.50 (s, 3H)	33.7		

† 5.32ppm for ^{1}H NMR, 54.0 ppm for ^{13}C NMR as internal reference

Figure S2. ^{1}H NMR spectrum of staurosporine (**1**) in CD_2Cl_2 (600 MHz, 298 K)

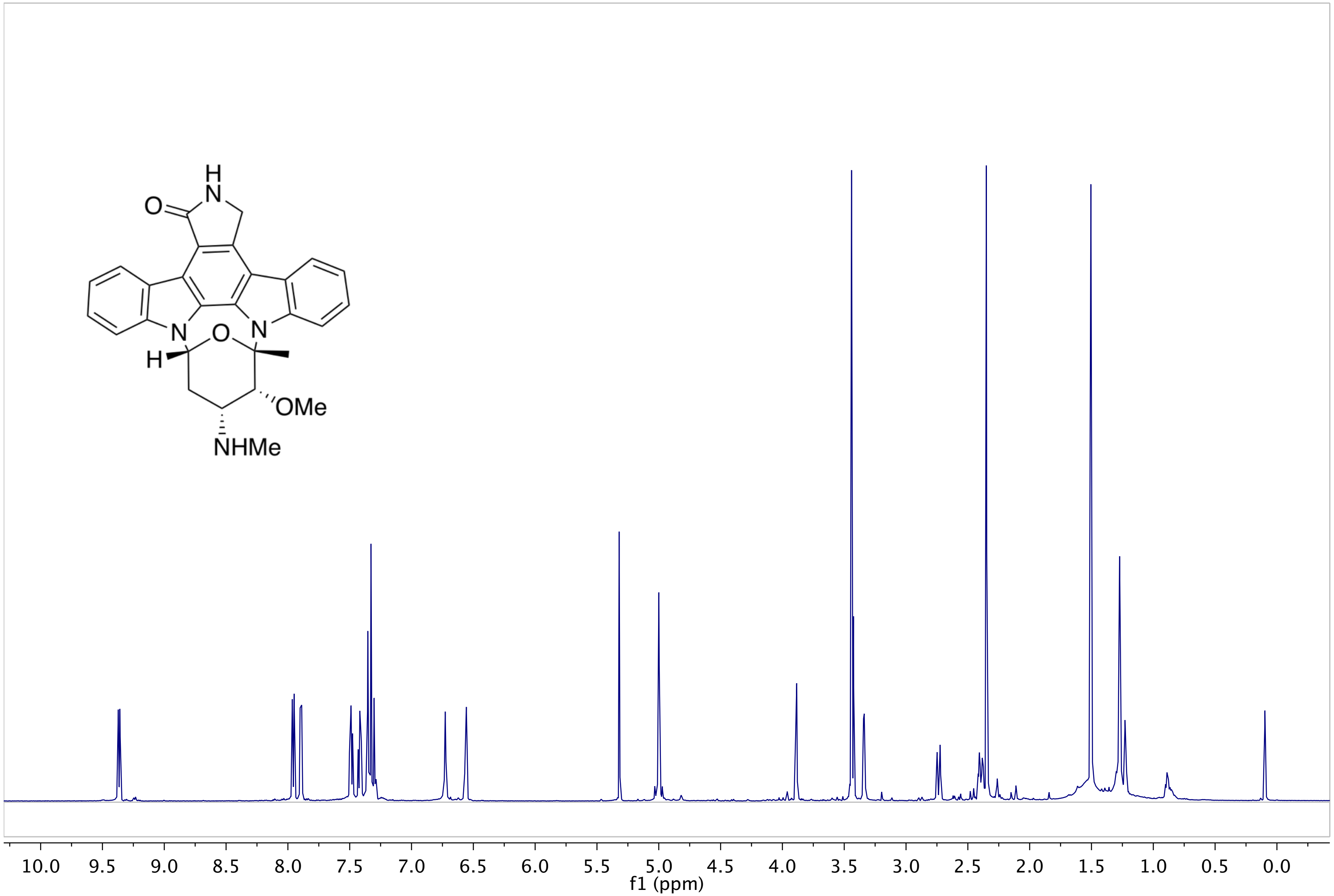

Figure S3. ^{13}C NMR spectrum of staurosporine (**1**) in CD_2Cl_2 (150 MHz, 298 K)

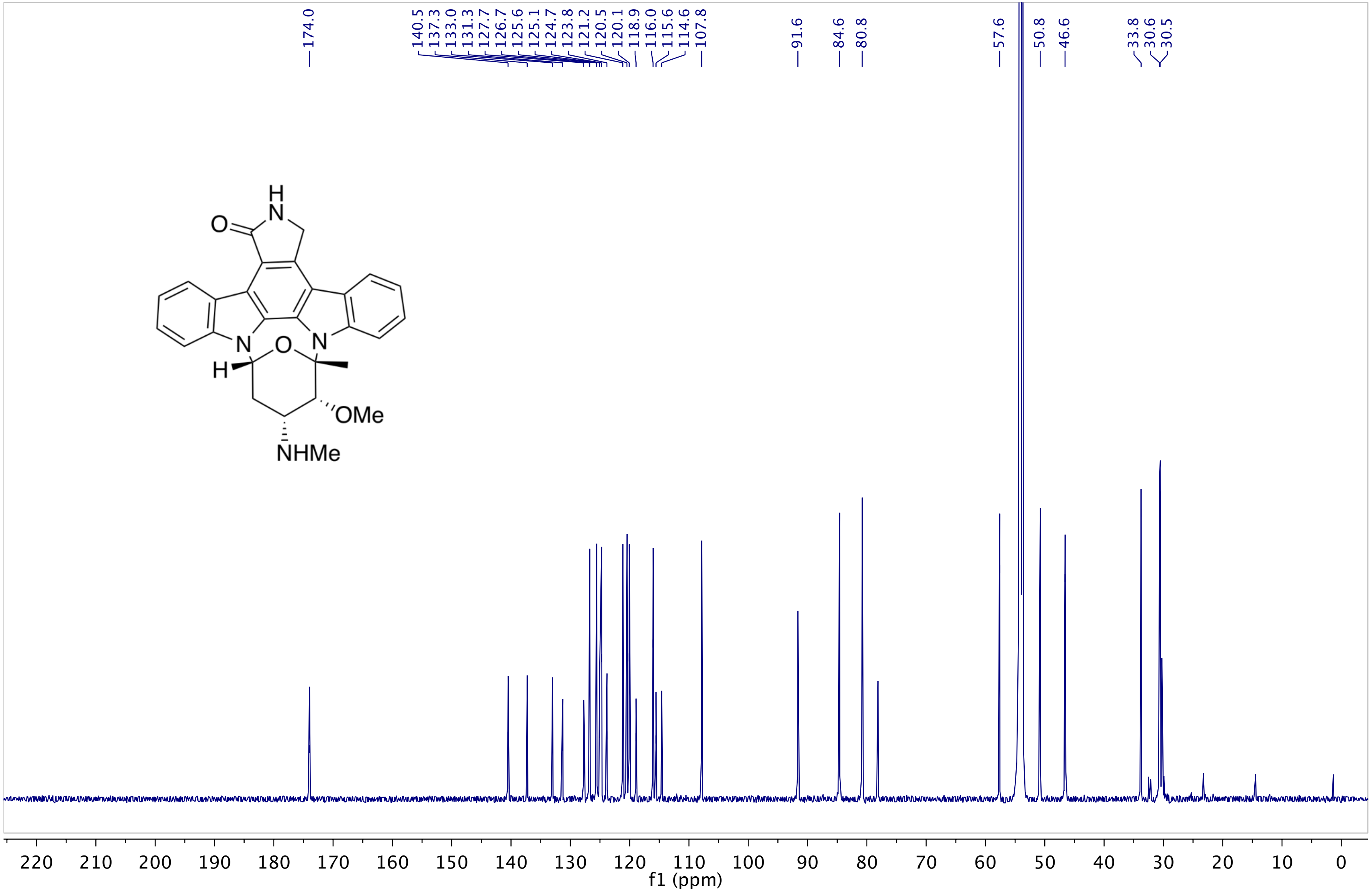

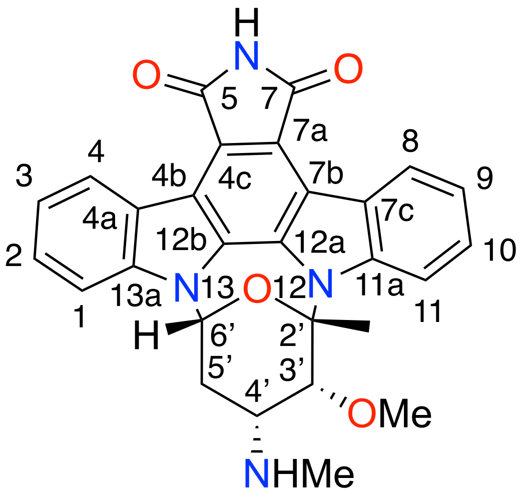

7-Oxostaurosporine (**2**)

Yellow powder, $[\alpha]_D^{20}$ +32.0° (*c* 0.1, $CHCl_3$)
UV ($CHCl_3$) λ_{max} (log ε) 319 (4.31) nm
IR υ_{max} 2920, 2851, 1713, 1568, 1463 and 1318 cm^{-1}
HRESIMS m/z 481.1875 $[M+H]^+$, calc. 481.1876 for $C_{28}H_{25}N_4O_4$

Figure S4. Chemical structure, atom carbon position numbers and physical data for 7-oxostaurosporine (**2**).

Table S2. NMR data for 7-oxostaurosporine (**2**) in CD_2Cl_2 (600 MHz, 298 K)

Carbon	δ^1H	$\delta^{13}C$	HMBC	ROESY
**1**	7.40 (d, *J*= 7.9 Hz, 1H)	108.4	3, 4a	6’
**2**	7.59 (t, *J* = 6.7 Hz, 1H)	127.2	4, 13a	
**3**	7.42 (t, *J*= 6.9 Hz, 1H)	121.2	1, 4a	
**4**	9.19 (d, *J* = 7.9 Hz, 1H)	126.3	2, 4b, 13a	
**4a**	-	122.7		
**4b**	-	115.8		
**4c**	-	119.8[b]		
**5**	-	170.6[a]		
**7**	-	170.4[a]		
**7a**	-	122.7[b]		
**7b**	-	117.4		
**7c**	-	124.1		
**8**	9.28 (d, *J*= 7.4 Hz, 1H)	125.5	7b, 10, 11a	
**9**	7.34 (dt, *J*= 7.1, 0.8, 1H)	120.9	7c, 11	
**10**	7.48 (t, *J*= 7.1, 1H)	126.5	8, 11a	
**11**	7.96 (br d, *J* = 8.6 Hz, 1H)	115.8	7c, 9	2'-CH_3
**11a**	-	141.6		
**12a**	**-**	132.3[b]		
**12b**	-	131.4[b]		
**13a**	**-**	138.6		
**2'**	-	91.7		
**3'**	3.90 (d, *J* = 3.7 Hz, 1H)	84.6	3'-OCH_3	
**4'**	3.37 (q, *J* = 3.6 Hz, 1H)	50.6	2', 4'-$NHCH_3$	
**5'α**	2.41 (ddd, *J* = 15.0, 5.6, 3.5 Hz, 1H)	30.2		
**5'β**	2.77 (ddd, *J* = 15.0, 3.7, 1.2 Hz, 1H)	30.2		
**6'**	6.55 (dd, *J* = 5.6, 1.2 Hz, 1H)	80.7	2', 4', 12b	
**2'-CH_3**	2.37 (s, 3H)	30.7	2', 3'	
**3'-OCH_3**	3.47 (s, 3H)	57.6	3'	
**4'-NCH_3**	1.50 (s, 3H)	33.7	4'	

† 5.32 ppm for ^1H, 54.0 ppm for ^{13}C as internal reference.
[a] Values with the same superscript are interchangeable in the same column.
[b] Established by comparison from NMR data of 7-oxostaurosporine derivatives reported from Jiménez *et al* 2012 (*Mar Drugs*, 10, 1092-1102).

Figure S5. ^{1}H NMR spectrum of 7-oxostaurosporine (**2**) in CD_2Cl_2 (600 MHz, 298 K)

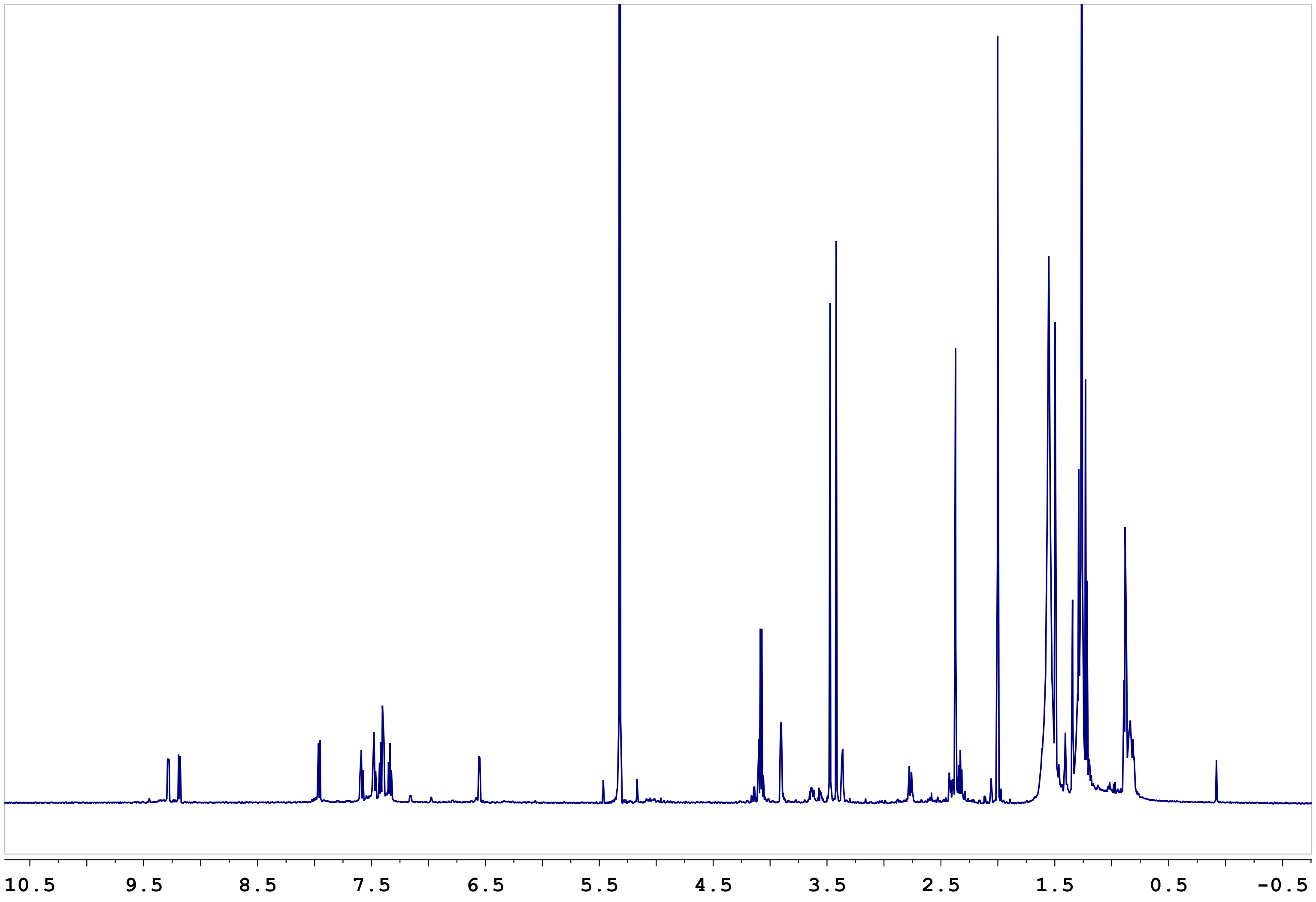

Figure S6. ^{13}C NMR spectrum of 7-oxostaurosporine (**2**) in CD_2Cl_2 (150 MHz, 298 K)

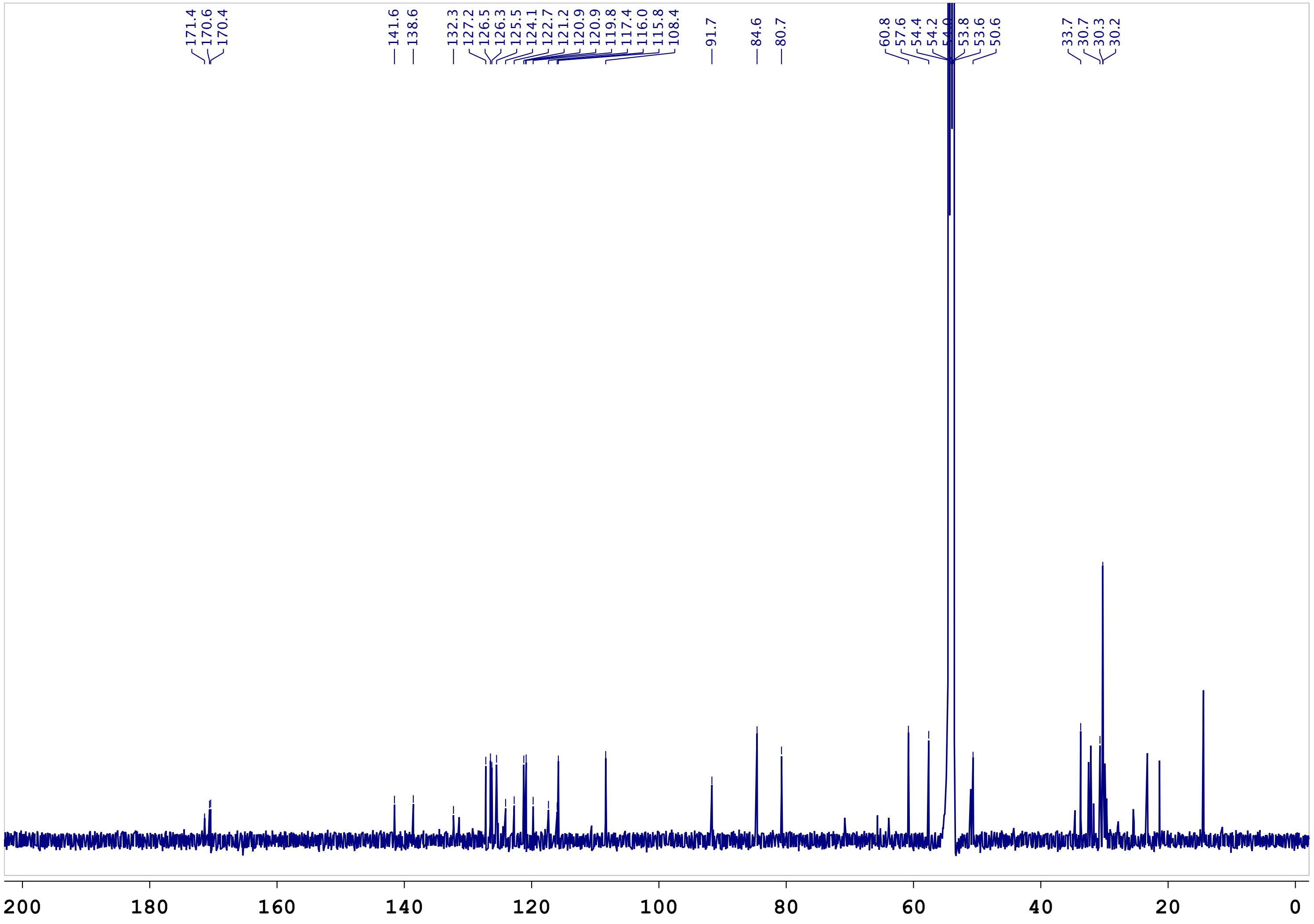

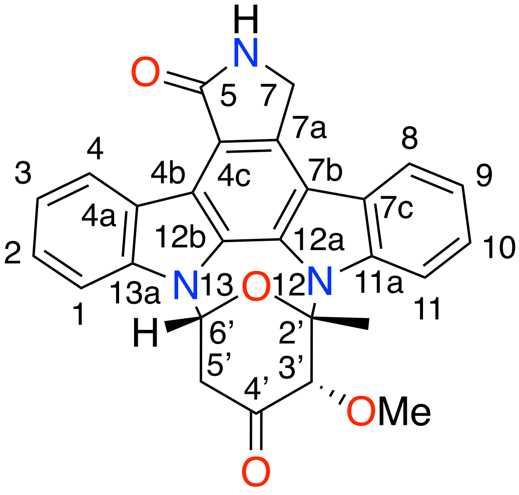

4'-Demethylamino-
4'-oxostaurosporine (**3**)

Yellow powder, $[\alpha]_D^{20}$ 22° (c 0.12, $CHCl_3$)
UV ($CHCl_3$) λ_{max} (log ε) 293 (4.36) nm
IR υ_{max} 2922, 2853, 2362, 1682, 1456 and 1317 cm−1
HRESIMS m/z 474.1425 $[M+Na]^+$, calc: 474.1430 for $C_{27}H_{21}N_3O_4Na$

Figure S7. Chemical structure, atom carbon position numbers and physical data for 4'-demethylamino-4'-oxostaurosporine (**3**).

Table S3. NMR data for 4'-demethylamino-4'-oxostaurosporine (**3**) in CD_2Cl_2 (600 MHz, 298 K)

Carbon	δ^1H	$\delta^{13}C$	HMBC	ROESY
**1**	7.33 (m, 1H)	108.2		6'
**2**	7.50 (t, *J*= 7.8, 1H)	126.4	4, 13a	
**3**	7.35 (t, *J*= 7.8, 1H)	121.2[a]	4a	
**4**	9.30 (d, *J*= 8.2, 1H)	127.1	2, 13a, 4b	
**4a**	-	121.2[a]		
**4b**	-	117.1		
**4c**	-	119.6		
**5**	-	173.3[‡]		
**7**	4.94 (s, 2H)	46.5	5, 4c, 7a, 7b	
**7a**	-	133.5		
**7b**	-	115.8		
**7c**	-	125.1		
**8**	7.87 (d, *J*= 7.8, 1H)	121.5	11a	
**9**	7.35 (t, *J*= 7.8, 1H)	121.2[a]	7c	
**10**	7.46 (t, *J*= 7.8, 1.4,1H)	125.6	8	
**11**	7.97 (d, *J* = 8.2, 1H)	116.3	7c	2'-CH_3
**11a**	-	140.9		
**12b**		128.3[‡]		
**12a**	-	124.4		
**13a**	-	136.9		
**2'**	-	100.1		
**3'**	4.59 (d, *J*= 1.0 Hz, 1H)	90.0	2', 4' (J^2), 3'-OCH_3, 2'-CH_3	2'-CH_3, 5'a, 3'-OCH_3
**4'-CO**	-	199.2[‡]		
**5'a**	3.66 (ddt, *J*= 0.9, 6.8, 14.2, 1H)	46.4	3', 6', 4'CO	
**5'b**	2.94 (dd, *J*= 0.9, 14.2, 1H)	46.4	4'CO	
**6'**	7.11 (dd, *J* = 6.8, 0.9, 1H)	85.0	2', 4', 5'a, 12b	
**2'-CH_3**	2.60 (s, 3H)	30.2	4' (J^4), 6', 2' (J^2)	
**3'-OCH_3**	3.52 (s, 3H)	60.1		

[†] 5.32ppm for ^1H, 54.0 ppm for ^{13}C as internal reference
[a] Values with the same superscript are interchangeable in the same column
[‡] Chemical shift assigned from HMBC experiment and by comparison with Cai Y. et al., 1996 (*J. Antibiot*. 1996, 49, 519–526)

Figure S8. ^{1}H NMR spectrum of 4'-demethylamino-4'-oxostaurosporine (**3**) in CD_2Cl_2 (600 MHz, 298 K)

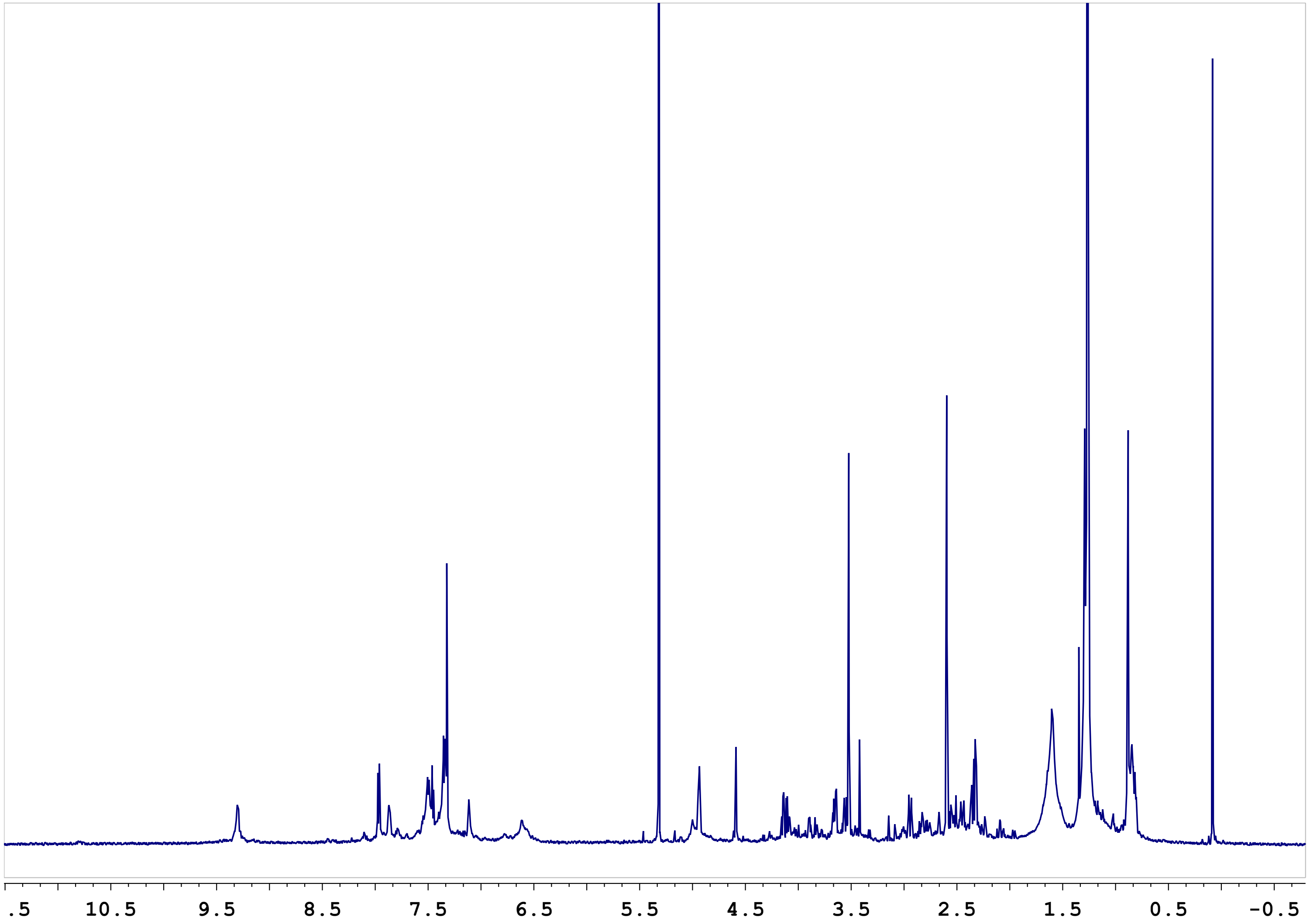

Figure S9. ^{13}C NMR spectrum of 4'-demethylamino-4'-oxostaurosporine (**3**) in CD_2Cl_2 (150 MHz, 298 K)

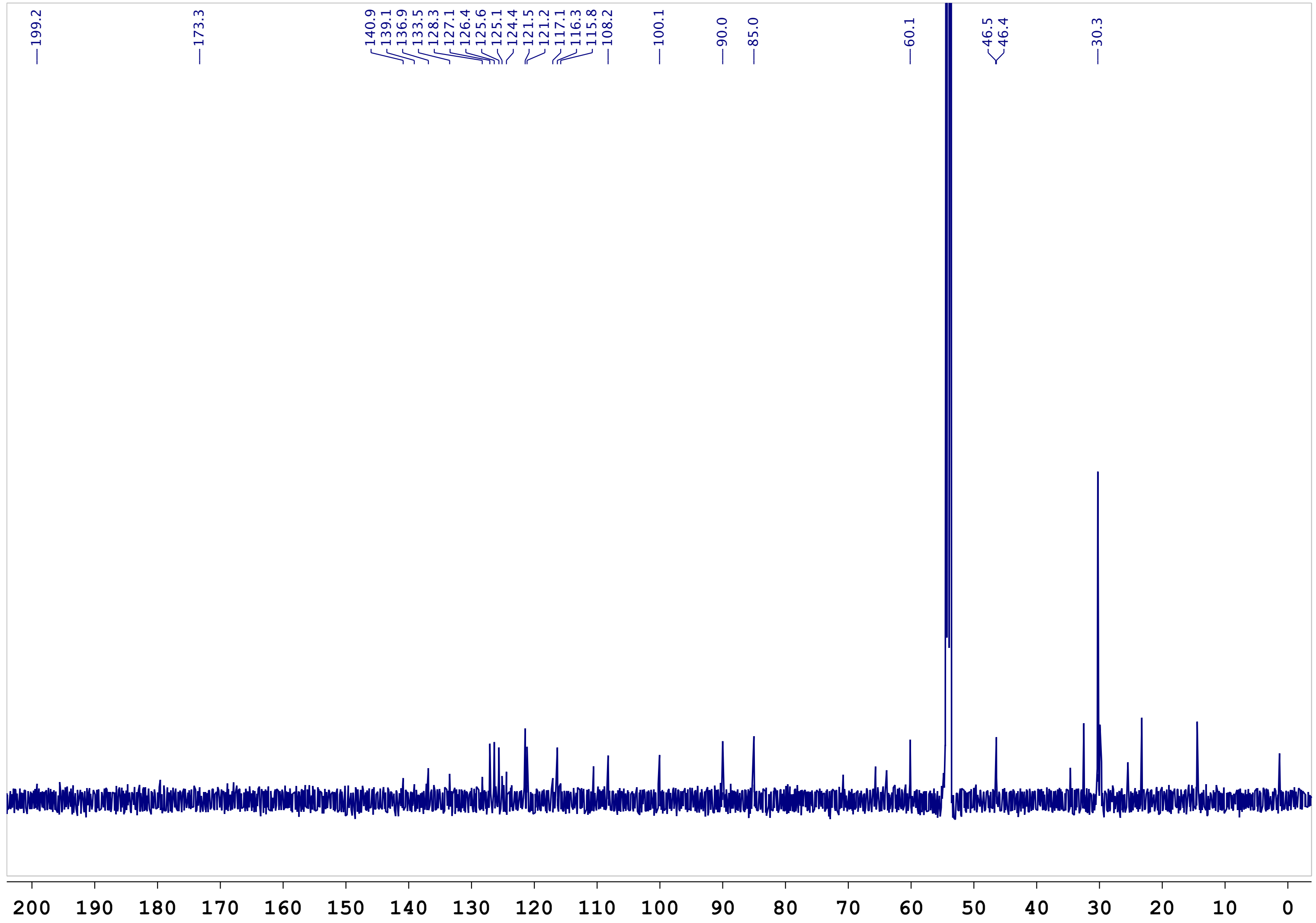

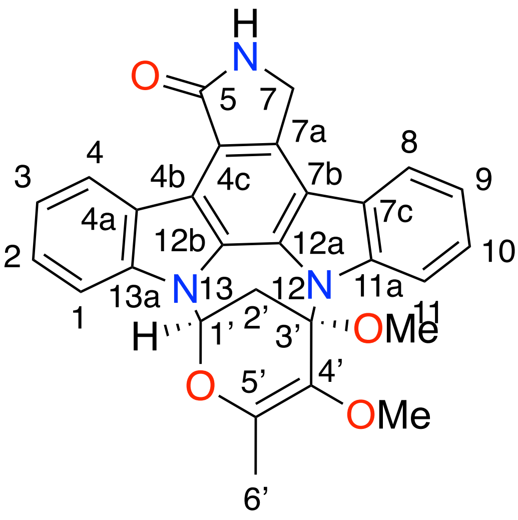

Streptocarbazol B (SCZ B, **4**)

Pale yellow powder, [a]$_D^{20}$ -27° (*c* 0.09, $CHCl_3$)
UV ($CHCl_3$) λ_{max} (log ε) 291 (4.35) nm
IR υ_{max} 2928, 1682 and 1456 cm^{-1}
HRESIMS m/z 488.1513 $[M+Na]^+$, calc: 488.1586 for $C_{28}H_{23}N_3O_4Na$

Figure S10. Chemical structure, atom carbon position numbers and physical data for streptocarbazole B (SCZ B, **4**)

Table S4. NMR data for streptocarbazole B (SCZ B, **4**) in CD_2Cl_2 (600 MHz, 298 K)

Carbon	δ^{1}H	δ ^{13}C	HMBC	ROESY
**1**	7.71 (d, *J* = 8.2 Hz, 1H)	109.5	4a	1'
**2**	7.57 (t, *J*= 7.6 Hz, 1H)	126.5	13a	
**3**	7.40 (t, *J*=7.4Hz, 1H)	121.6	1	
**4**	9.41 (d, *J* = 7.9 Hz, 1H)	126.6	2, 13a	
**4a**	-	124.05		
**4b**	-	117.8		
**4c**	-	119.5		
**5**	-	173.1		
**6**	6.23 (NH)	-	4c, 7, 7a	
**7**	5.03 (dd, *J* = 2.2, 1.1 Hz, 2H)	46.2	4c, 7a	
**7a**	-	134.1		
**7b**	-	116.8		
**7c**	-	124.09		
**8**	7.94 (d, *J* = 8.0 Hz, 1H)	121.2	7b, 10, 11a	7, 9
**9**	7.36 (*J*= 7.5 Hz, 1H)	121.3	11	
**10**	7.53 (t, *J*= 7.8 Hz, 1H)	126.5	8, 11a	11
**11**	8.30 (d, *J* = 8.4 Hz, 1H)	115.8	7c, 9	3'-OCH_3
**11a**	-	141.5		
**12a**	-	133.6		
**12b**	-	126.1		
**13a**	-	140.1		
**1'**	6.75 (dd, *J* = 5.8, 1.6 Hz, 1H)	78.2	3', 5', 12b	1
**2'A**	3.43 (dd, *J* = 14.8, 5.6 Hz, 2H)	38.8	1', 3', 4'	
**2'B**	3.37 (dd, *J* = 14.8, 1.6 Hz, 1H)	38.8	3', 4'	
**3'**	-	88.0		
**4'**	-	133.7		
**5'**	-	144.1		
**6'-CH_3**	1.68 (s, 3H)	14.8	4', 5'	
**3'-OCH_3**	3.62 (s, 3H)	51.7		11
**4'-OCH_3**	3.11 (s, 3H)	62.3	4'	6'-CH_3

† 5.32ppm for ^{1}H, 54.0 ppm for ^{13}C as internal reference

Figure S11. ^{1}H NMR spectrum of streptocarbazole B (SCZ B, **4**) in CD_2Cl_2 (600 MHz, 298 K)

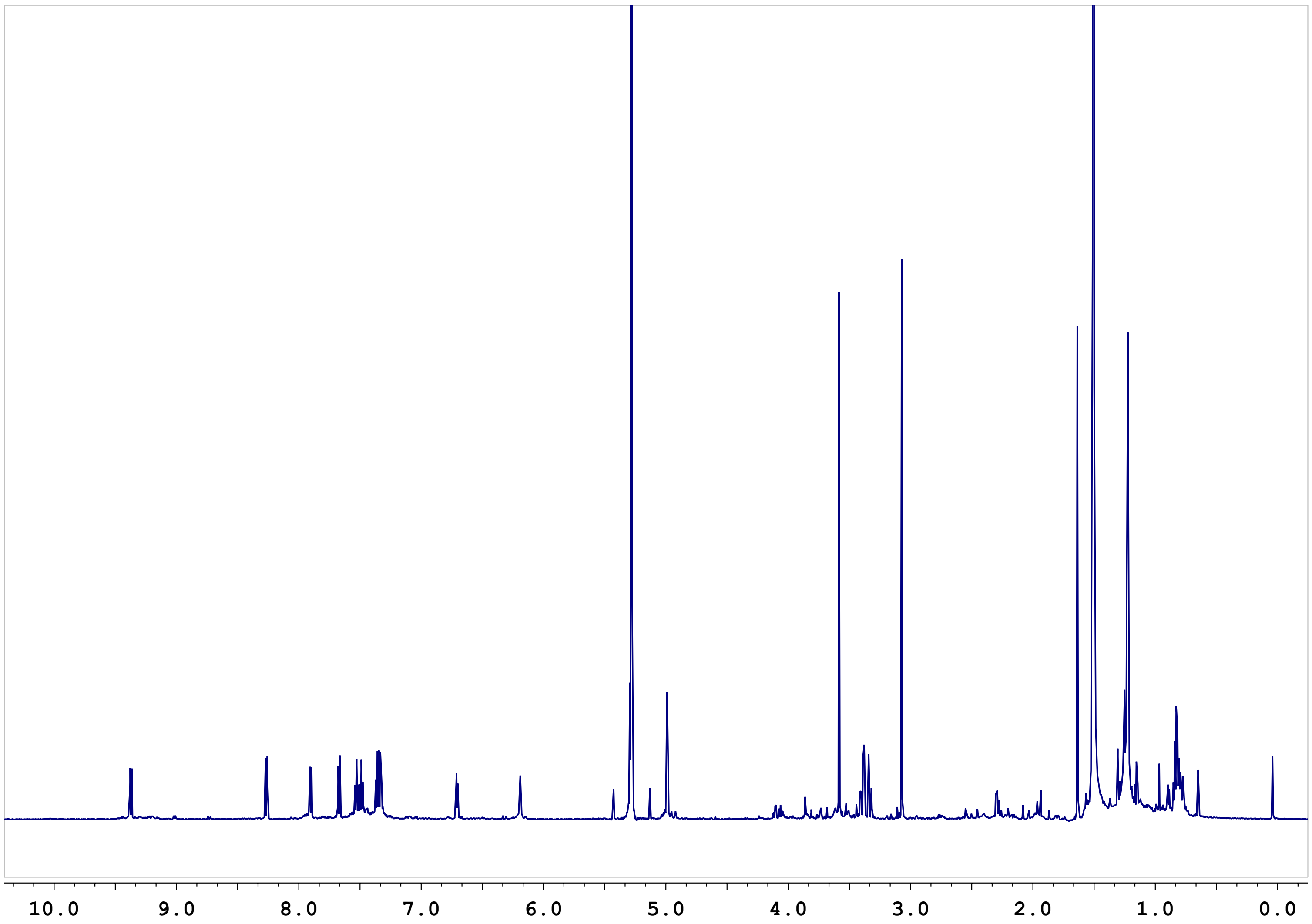

Figure S12. ^{13}C NMR spectrum of streptocarbazole B (SCZ B, **4**) in CD_2Cl_2 (150 MHz, 298 K)

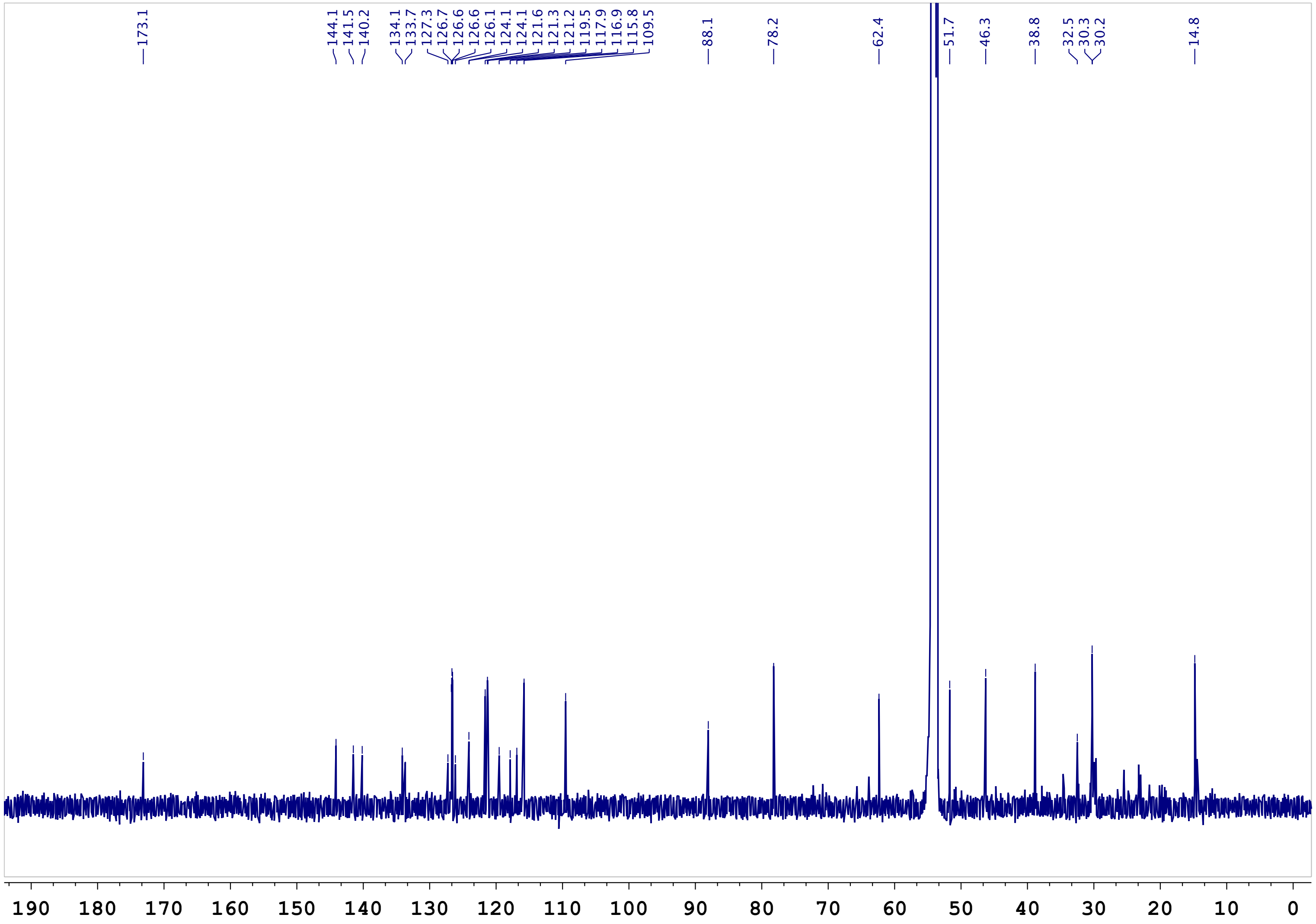

Supplement: Supplementary file 1 [file marinedrugs-17-00588-s001.pdf]
